# Evaluating and predicting net energy value of wheat and wheat bran for broiler chickens

**DOI:** 10.5713/ab.21.0501

**Published:** 2022-04-29

**Authors:** Ran Ning, Zichen Cheng, Xingbo Liu, Zhibin Ban, Yuming Guo, Wei Nie

**Affiliations:** 1 State Key Laboratory of Animal Nutrition, College of Animal Science and Technology, China Agricultural University, Beijing 100193, China; 2 Laboratory of Animal Nutrition Metabolism, Jilin Academy of Agricultural Sciences Gongzhuling, Jilin 136100, China

**Keywords:** Broiler, Net Energy, Predication Equation, Wheat, Wheat Bran

## Abstract

**Objective:**

It is crucial to accurately determine the net energy (NE) values of feed ingredients because the NE system is expected to be applied to the formulation of broilers feed. The NE values of 5 wheat and 5 wheat brans were determined in 12-to 14-day old Arbor Acres (AA) broilers with substitution method and indirect calorimetry method.

**Methods:**

A total of 12 diets, including 2 reference diets (REF) and 10 test diets (5 wheat diets and 5 wheat bran diets) containing 30% of test ingredients, were randomly fed to 864 male AA birds with 6 replicates of 12 birds per treatment. These birds were used to determine metabolizable energy (ME) (8 birds per replicate) in the chicken house and NE (4 birds per replicate) in the chamber respectively at the same time. After a 4-d dietary and environment adaptation period, growth performance, energy values, energy balance and energy utilization were measured during the following 3 d. Multiple linear regression analyses were further performed to generate prediction equations for NE values based on the chemical components and ME values. The NE prediction equation were also validated on another wheat diet and another wheat bran diet with high correlation (r = 0.98, r = 0.75).

**Results:**

The NE values of 5 wheat and 5 wheat bran samples are 9.34, 10.02, 10.27, 11.33, and 10.49 MJ/kg, and 5.37, 5.17, 4.87, 5.06, and 4.88 MJ/kg DM, respectively. The equation with the best fit were NE = 1.968AME−0.411×ADF−14.227 (for wheat) and NE = −0.382×CF −0.362×CP−0.244×ADF+20.870 (for wheat bran).

**Conclusion:**

The mean NE values of wheat and wheat bran are 10.29 and 5.07 MJ/kg DM in AA broilers. The NE values of ingredients could be predicted by their chemical composition and energy value with good fitness.

## INTRODUCTION

Poultry needs to metabolize energy-yielding nutrients to maintain life and production. Wheat is the main cereal ingredient used in commercial broiler diets in European and Oceania countries [1]. Meanwhile, wheat bran, the byproduct of wheat, was recently reported that had a prebiotic effect [2]. Poultry diets formulation could be more profitable if we accurately measured the energy values of these common ingredients. Metabolizable energy (ME) system including apparent metabolizable energy (AME), true metabolizable energy (TME) and AME corrected to zero or 50% nitrogen retention (AMEn, AMEs) [3] is commonly used to formulate diets in poultry industry. Azhar et al [1] used the total collection method to measure the AME of different wheat samples in 19- to 21-day-old Ross 308 broilers with a mean value of 14.21 MJ/kg dry matter (DM). Also, the indicator method was used by Karunaratne et al [4] to determine the AMEn which ranged from 13.40 MJ/kg to 14.27 MJ/kg (90% DM) in 0- to 21-day-old Ross 308 broilers. Net energy (NE) system recently used in pig and dairy industries can consider energy distribution from a more commercial perspective compared with ME system. The advantages are due to the consideration of heat increment (HI) that is the heat produced when energy is more than requirements for maintenance of livestock. The energy utilization radio (NE:AME) conducted in broilers by Wu et al [3] for protein, fat and starch were 52%, 85%, and 79%, respectively. The researchers simultaneously generated NE prediction equation and predicted the NE values of wheat (11.11 MJ/kg DM) and wheat bran (6.05 MJ/kg DM) for broilers. The similar tendency (fiber≤protein<starch<fat) of the efficiency ratio was also presented in the experimental results of laying hens obtained by Carre et al [5]. The observations indicated that HI of these nutrients in broilers was also different which could illustrate the necessity and importance to formulate poultry diets based on NE.

The direct method, reference diet (REF) substitution method and prediction equation method are commonly used to determine the energy value of ingredients. As birds cannot digest fiber well and wheat and wheat bran are rich in fiber, we generally choose the REF substitution method when carrying out metabolism experiments *in vivo*. Ignoring the interaction between the ingredients, a substitution ratio is used to calculate the corresponding energy values of ingredients through those diets containing test ingredients. The heat production (HP) including fasting heat production (FHP) is frequently determined by the comparative slaughter method or indirect calorimetry method [6]. However, the feed intake (FI) of broilers increases with age. The FI and excreta measurement of broilers provided with ad-libitum feed in the respiratory calorimetry chambers could not accurately reflect the relationship between AME and NE. It may offer more precise data if we determine the ME of broilers with certain fasting time and HP of similar broilers with ad-libitum feed. Although energy is always the focus of feed studies, the research on the net energy values of wheat and wheat bran is rare, especially in the starter phase of the broilers.

The objective of present study was to measure the NE values of 5 wheat and 5 wheat bran ingredients in commercial broilers and to generate NE prediction equations from the chemical composition and ME values. Also, additional wheat and wheat bran diets were used to validate the resulting equations.

## MATERIALS AND METHODS

### Ethical statement

All experiments were conducted at the Animal Ethics Committee of the Jilin Academy of Agricultural Sciences. The procedures were performed according to the guidelines for animal experiments set by the National Institute of Animal Health, China (SYXK20190059).

### Wheat, wheat bran and diets

The 6 wheat samples and 6 wheat bran samples were obtained from different areas (Shandong, Henan, Jiangsu, Liaoning, Jilin, Anhui) in China. The chemical composition of the 12 ingredients is shown in Table 1 and 2. The formula of reference and test diets are shown in Table 3 and the nutrient level of diets is shown in Table 4. The composition of a single diet in Experiment 1 was the same as the composition of the REF in Experiment 2, except for the varieties of some ingredients such as corn, oil, etc. In Experiment 2, the REF was formulated with corn, soybean meal (SBM), corn gluten meal, peanut meal, soybean oil, dried distillers grains with solubles (DDGS), and the test diets used either wheat or wheat bran to replace 30% (air-dry basis) of the REF [7]. The diets used for the validation of prediction equations in experiment 3 was the same as the composition of the test diet in experiment 2, except for the varieties of the wheat or wheat bran samples. All ingredients were supplied from Wellhope and analyzed for nutrient content by Centre Testing International Group Co., Ltd.

### Birds and feeding management

Forty-eight (Experiment 1), 864 (Experiment 2), or 144 (Experiment 3) male Arbor Acres (AA) broilers at 8 days were selected from the local farms. In Experiment 1, broilers were randomly allocated to 12 chambers with the same diet in a single run. In Experiment 2, broilers were obtained in 6 times (144 broilers per time) and randomly allocated to 12 treatments (2 REFs and 10 test diets). A total of 144 broilers of two treatments were used per time. A total of 96 broilers were housed in the chicken house (8 birds per cage) and 48 broilers were housed in the calorimetry chambers (4 birds per chamber). Experiment 3 was performed with two diets (one wheat diet and one wheat bran diet), 6 replicates in a single run with 12 chambers. Broilers were reared following the AA recommendations [8] with 20 h of light and 4 h of darkness at all times. For feed and environment adaption, birds were fed the test diets and feed and water were provided ad libitum in acclimation period and the calorimetry chambers lids were open with air pumps running. The room controlled the environment climate by air-condition at 27°C to 31°C during the formal test period (from day 12 to day 14).

### Calorimetry chambers and measurements of gas concentrations

The equipment is composed of sensor and analyzer, respiration chamber, air conditioner and heater and so on. Its principle can refer to the description of Van Milgen et al [9]. Twelve open-circuit respiration chambers of approximately 0.54 m^3^ with a design similar to that of Liu et al [10] and Liu et al [11] were used in this study. There were 304 stainless steel chambers and the lids were made up with plexiglass. Each chamber has gas circulation, air conditioner, heater, dehumidification, sensors, and other equipment. The temperature can be regulated from 16°C to 45°C with resolution of 0.1°C; the humidity can be regulated from 30% to 95% with resolution of 1.0% and flow speed can be regulated from 4 to 40 L/min by the float flow meter. They can monitor the O_2_ consumption and CO_2_ production of different chambers in real time online, and automatically calculate the respiratory quotient (RQ). The measuring range of carbon dioxide and oxygen is 0–1% and 0–25% respectively; the accuracy of the analytical instrument is 0.5% of the measuring range. Compared with the close-circuit calorimetry chamber, the one used in this study can maintain the atmospheric pressure environment, which more truly reflects the physiological state of birds. The tightness of the equipment depends on the water.

### Experimental design

Experiment 1 was conducted to determine NE variation between 12 chambers. The final experiment was to examine the accuracy of the NE prediction equations generated from Experiment 2. For all the three experiments, broilers at 8 days of age with a similar initial body weight (BW) (close to 210 g) were randomly allotted to all diets. A total of 6 runs were conducted with 2 diets per run. For one diet, 8 birds per cage were selected for ME determination in the shed and 4 birds per chamber were used to measure the HP in the equipment. Birds at 8 days of age were adapted to corresponding diets and environment (shed or chamber) for 4 d. Broilers in the shed were fasted from 4:00 p.m. (day 11) to 9:00 a.m. (day 12) during the acclimation period. From 12 days old to 14 days old, birds were still fed the corresponding diet and were provided with water ad libitum. Then, they were fasted from 4:00 p.m. (day 14) to 9:00 a.m. (day 15) for the accuracy of the excreta collection [12]. Those broilers used to measure HP were not fasted during the formal test period, instead they were treated like those used to measure ME. Birds were all weighted at day 12 and day 15. The consumption of feed was also measured every day. The O_2_ consumption and CO_2_ production were measured for 3 consecutive days (from day 12 to 15) to calculate the HP.

### Samples collection and chemical analysis

The total excreta of each replicate were collected and sprayed with hydrochloric acid daily in the shed, and then they were pooled, dried, regained for 1 day and weighted. FI was measured by the total consumption of feed divided by 3 d. The feed and excreta samples were refrigerated (4°C) and were ground through a 40-mesh screen prior to chemical analysis. The gross energy (GE) of diets and excreta was determined by a bomb calorimeter (IKA-C3000, Staufen, Germany). Excreta and feed samples were analyzed for moisture (AOAC, 2000, method 934.01). Crude protein (CP) and nitrogen (N) content (AOAC, 2000; method 990.03) [13]. Ingredients were further analyzed for ether extract (EE) that was extracted with 40°C to 60°C petroleum ether by ether extraction method (AOAC, 2012; 920.39) and for crude fiber (CF) that was also using standard analytical procedures (AOAC, 2012; 962.09). Neutral detergent fiber (NDF) and acid detergent fiber (ADF) were determined using an Ankom220 Fiber Analyzer (Ankom Technology, NY, USA) (AOAC, 2012; 973.18).

### Calculations

The average daily gain (ADG) was calculated as total weight gain divided by the corresponding days (3 d) and the number of birds (4 or 8) (g/d). The average body weight (ABW) of was the mean of the initial and final weight (g). Feed conversion ratio (FCR) was calculated as the ratio of FI to ADG (g/g). AME were determined by the total collection method previously described by Bourdillon et al [14] with modifications. 34.39 MJ/kg of N was used as the correction factor of AMEn [15]. AME intake (AMEI) was calculated as the ratio of different between the GE of FI and the GE of excreta to FI. Total heat production (THP) was calculated by gas concentrations previously described by Wu et al [3] refer to the equation1: THP (MJ/kg) = 16.1753×O_2_ consumed (L)+ 5.0208×CO_2_ produced (L). The HI was calculated by subtracting FHP (450 kJ/BW^0.70^) that was estimated by Noblet et al [16] from total HP. The RQ as corresponds to the ratio of liters of CO_2_ expired to liters of O_2_ consumed [17]. NE intake (NEI) was calculated by subtracting HI from AMEI. The difference between N intake and N excreta was the total nitrogen retained (TNR). Retention of energy (RE), RE as protein and RE as fat were calculated according to the equation 2, 3, 4:

RE (MJ/kg) = MEI−THP (equation 2), RE as protein (MJ/kg) = TNR×6.25×5.7 (6.25 is the protein equivalent of 1 g nitrogen, and 5.7 is the energy equivalent of 1 g protein (MJ/kg/d)) (equation 3), RE as fat (MJ/kg) = RE−RE as protein (equation 4). Further, the AME value and NE value of the wheat and wheat bran were determined by the substitution method [7]. ME_ingredient_ (MJ/kg) = (ME_test_− ME_reference_×a%)/b% NE_ingredient_ (MJ/kg) = (NE_test_−NE_reference_×a%)/b% (in this study a = 67.58, is the inclusion level of energy-yielding ingredients including grains, soybean meal, corn gluten meal, peanut meal, soybean oil, DDGS and amino acids from the REF in the test diet and in this study b = 31.32, is the substitution level of the ingredient in the test diet).

### Statistical analyses

The data were analyzed statistically via one-way analysis of variance using the general linear model procedure in SPSS 25.0 [18]. Significant differences among treatment means were determined by using Duncan’s multiple-range test. Differences were considered statistically significant at p<0.05.

## RESULTS

### Growth performance and energy

The low variability (as RSD) for the THP, NE, and other measured parameters indicate the uniformity of the test system (Tables 5, 6). The effects of wheat or wheat bran replacement on performance and energy value of broilers are also presented in Table 5 and 6. The growth performance data in Table 5 shows that test wheat diet 2 significantly improved FI and HI and test wheat diet 2 and 3 increase AM intake, NEI and RE (p<0.05). All test wheat bran diets significantly decrease NEI and RE (p<0.05). However, inclusion of wheat did not show any impact on the energy values and efficiency (Tables 5, 6). The energy value and efficiency of GE utilization for AME or AMEn in AA chicken were affected by wheat bran, but not wheat (p<0.01).

### Energy and energy utilization values of ingredient

As indicated in Table 7, the AME values of wheat ranged from 12.93 to 13.75 MJ/kg, the AMEn from 12.70 to 13.50, the NE from 9.34 to 11.33 (p>0.05). The values of wheat bran were obviously lower, the AME values of wheat bran ranged from 6.85 to 7.72, the AMEn from 6.66 to 7.49, the NE from 4.88 to 5.37 (p>0.05) (Table 8). In addition, no differences were observed in the energy utilization of the five wheat samples and five wheat bran samples (p>0.05).

### Correlation analysis and prediction equation

Table 9 and Table 10 shows the correlation analysis between nutritional composition and energy contents of wheat or wheat bran. CF, NDF, and ADF of wheat had a close relation as we expected (p<0.01) (Table 9) while CF and ADF of wheat bran had no correlation to each other (Table 10). Interestingly, CP of wheat had no correlation with ADF or CF of wheat (p>0.05) (Table 9). Prediction equations for wheat and wheat bran NE values were developed by multiple stepwise regression against the ingredient nutrients and ME values (Table 11). There is no significant correlation between independent variables in each equation (p>0.05). Therefore, correlation analyses ensured the accuracy of regression equations to predict NE using chemical components. The first equation in Table 11 shows NE values of wheat to be positively related (1.968) to AME and negatively related (−0.411) to ADF as we expected. For the second equation in Table 11, CP, CF, and ADF was negatively related (−0.362, −0.382, −0.244) to NE values.

### Validation of net energy prediction equation

**Figure 1:**
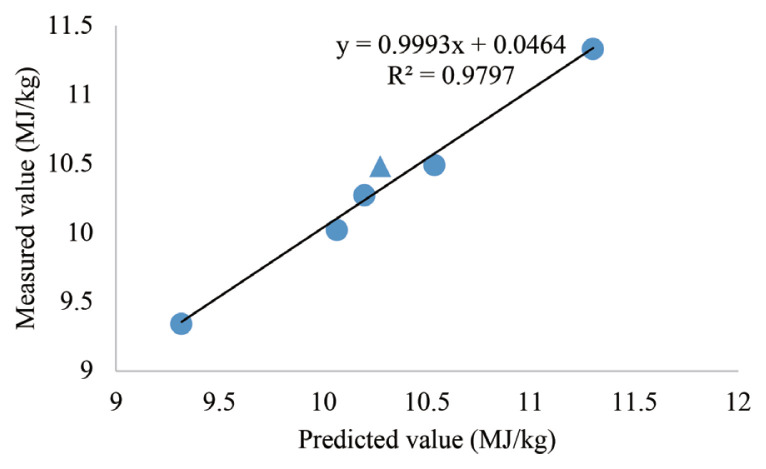
Comparisons between actual wheat NE values measured by indirect calorimetry and predicted wheat NE values estimated by regression equations were used to validate the accuracy of the NE prediction equation (the triangle indicates the validation value, the circle indicates the values measured in Experiment 2). NE, net energy.

**Figure 2:**
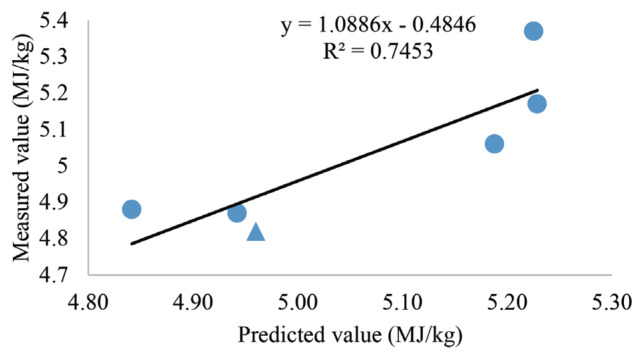
Comparisons between actual wheat bran NE values measured by indirect calorimetry and predicted wheat bran NE values estimated by regression equations were used to validate the accuracy of the NE prediction equation (the triangle indicates the validation value, the circle indicates the values measured in Experiment 2). NE, net energy.

Figure 1 and 2 shows the validation of prediction equations for the NE of wheat or wheat bran, respectively. The additional diets were formulated with the same ingredients that involved in the test diet, expect for the varieties of the test wheat or the test wheat bran. Open-circuit calorimetry chambers were used to measure the NE values of another wheat sample and another wheat bran sample. Then, they were used to compare with predicted NE values which were estimated by nutrient parameter and ME values of ingredient in the prediction equations. The liner regression coefficient (r = 0.98, r = 0.75) indicated that the predicted and measured NE values of wheat or wheat bran are close.

## DISCUSSION

The differences of proximate composition among 5 wheat and 5 wheat bran varieties may be caused by the source [1], season and other factors. Wheat bran contained a higher proportion of CP and therefore the wheat bran diets had a higher CP content compared with the wheat diet (Table 1, 2, 4).

In the present study, the substitution of 30% wheat increased the FI of broilers (p<0.05) when compared with REF. This may explain why some test wheat diets could improve the AM intake, NEI, HI, and RE (p<0.05). The observation is consistent with the previous results that wheat diets with 60.2% wheat resulted in better FI than corn diets [19]. This may be due to the GE of the wheat diets are lower than the REF (p>0.05). Inclusion of wheat bran showed no or negative effects on the growth performance in broiler starters (Table 6). Our results agreed with many studies on wheat bran or other cereals [20,21]. However, a previous study [22] showed a conflicting result that supplementation of 3% wheat bran caused the ADG of broilers to increase by 4.8% in the starter phase when compared with the control diet. The source and inclusion level [23] of fiber can be regarded as the influencing factors that caused the contradictory results. This may also explain why test wheat bran diets could improve the NEI and RE (p<0.05).

As shown in Table 5 and 6, dietary treatment did not change the AMEI and its partition for HP and RE (p>0.05). The HP, RE and RQ values of broilers fed wheat diets in the present study were similar to the corresponding values of 15-day broilers fed *ad libitum* feed intake reported by Liu et al [6]. REF with high energy values brought higher AMEI, NEI, and RE than those of test wheat bran diets though the differences were not significant. This was consistent with finding reported in a previous study that dietary energy concentration could significantly affect TME intake and RE [10]. REF and test diets containing 30% wheat had similar energy values which is possible due to the close AME values of corn and wheat ingredients (13.64 MJ/kg, 12.72 MJ/kg) according to the Tables of Feed Composition and Nutritive Values in China (30th edition) [24]. Moreover, the mean AME:GE (73%) and AMEn:GE (72%) ratios of REF in this study is comparable with those observed by Wu et al [3] and Liu et al [25] while the mean NE:AME (62%) and NE:AMEn (63%) ratios of REF are relatively lower than the values reported by other previous studies [3,5,6]. This may be due to the formulation differences between the diets in two studies. The results were supported by the previous observation that organs developed and matured with bird age [26]. Meanwhile, the method used to measure ME in this study considers that appropriate fasting may correspond to the amount of FI and excretion more accurately in a given time. The equipment used to measure gas exchange is either open-circuit or close-circuit. Therefore, the differences of energy utilization efficiency may also be caused by the determination differences. The ME and NE values of the test diets decreased as wheat bran was introduced into the diets, which might be similarly approved by the lower AME value of wheat bran than that of corn [24]. As we expected, the non-starch polysaccharide (NSP) of wheat and wheat bran negatively affects energy values and energy utilization values of diets containing them by increasing digesta viscosity and encapsulating nutrients [27,28]. Furthermore, Musigwa et al [29] suggested that the soluble NSP content was inversely related to the measurements of diets mentioned above.

The mean AME, AMEn, and NE values of wheat in our study are 13.24, 12.98, and 10.29 MJ/kg DM, and those of wheat bran in our study are 7.11, 6.91, and 5.07 MJ/kg DM. The AMEn values of wheat (mean is 13.87 MJ/kg) in 0- to 21-day-old broilers fed wheat-based diets contained 63.08% of test wheat samples measured by Karunaratne et al [4] were higher than the results we got in the study. The values obtained above in our study were also lower than the predicted AMEn and NE values of wheat soft (13.94, 11.11 MJ/kg) and wheat bran (7.69, 6.05 MJ/kg) in 25- to 28-day-old broilers fed balanced diets reported recently in Wu et al [3]. The nutrient utilization differences among broilers in different developmental phases might explain this. The digestion and absorption efficiency of anti-nutrients in poultry diets might promote the maturation of gut microbiota [30]. The substitution method used in this study replaced energy-yielding ingredients with the test ingredient and the a% and b% in calculation equation were in the DM basis. In addition, diets containing wheat or wheat bran in previous study of Wu et al [3] were supplemented with enzymes. It was confirmed by previous study that enzyme-supplemented diets could improve HP, NEp, and Fat_RE_ [27,31,32]. Moreover, the fasting procedure in the AME measurement rarely occurred in previous studies. Therefore, the replaced feed formulation ignored the balance in the nutrient profile of test diets and the experimental procedures were different resulting in the differences between the studies. Also, the wheat or wheat bran varieties were from a different country. Wu et al [3] indicated that the AME:GE and AMEn:GE are correlated to CP (−0.52, −0.57) and EE (0.70, 0.69). The conclusion approved that the ratios in the current study (0.720, 0.709) were 9% lower than the values reported by Azhar et al [1] (0.790, 0.769). Wu et al [3] demonstrated that the NE:AMEn ratios (0.797, 0.786) of wheat soft and wheat bran calculated by equation in finisher-phase broilers fed balanced diets were higher than our results. This could also be supported by the negative correlation (−0.18) between NE:AMEn and CP reported by Wu et al [3], and the higher CP content of test wheat bran compared with the reference cereals mentioned above [24,33].

Stepwise linear regression equations for prediction of NE using AME and content of ingredients composition have been generated in Table 11. According to the equations, the NE values for broilers could be predicted from ME and ingredient chemical composition. In terms of wheat, ADF increase the NE values. CP and CF showed similar negative tendency in wheat bran NE prediction equations, while ST was positively related to NE. This is consistent with the previous result [34] that NE prediction equations generated from dietary nutrients where fiber (NDF) was a negative predictor. However, EE was positively related to NE [34] and fiber content was absent [3] in prediction equation as inferred from the study by others. It cannot be excluded that the enzyme supplement led to the absence of fiber effect. Hence, the inclusion of CF, ADF, and other fiber content into equations may improve the efficiency and accuracy of NE prediction [35]. Also, we compared the NE value measured by experiment (2,3) and NE predicted based on different equation as shown in Figure 1 and 2 which indicated that the regression equations generated in our study were relatively accurate.

## CONCLUSION

The NE values of the 5 wheat and 5 wheat bran samples were measured by substitution method and indirect calorimetry method in AA broilers. The mean NE values of wheat and wheat bran are 8.39 and 3.84 MJ/kg DM. In addition, the respective prediction equations were generated to predict NE values of ingredients from ingredient chemical composition. The commercial value and accuracy of results still need further validation experiments. These outcomes could provide references for NE database of cereals and diet formulations in poultry industry.

## Figures and Tables

**Table 1:** Chemical composition of 5 wheat samples (%, DM basis)

Items	Experiment 2					Experiment 3	Mean	CV (%)

	Wheat 1	Wheat 2	Wheat 3	Wheat 4	Wheat 5			
DM	88.60	91.20	89.30	83.20	85.00	87.80	87.52	3.35
CP	14.75	14.01	14.78	14.53	14.21	14.45	14.46	2.09
EE	1.92	1.75	2.02	2.04	2.24	2.05	2.00	8.08
CF	2.71	2.30	2.13	2.28	2.82	3.53	2.63	19.65
NDF	39.39	35.31	33.93	40.26	39.18	38.15	18.90	50.20
ADF	4.63	3.62	3.25	3.73	3.53	4.78	3.92	16.00
ST (g/kg)	646.73	627.19	665.17	682.69	672.94	602.51	649.54	4.67

CV, coefficient of variation; DM, dry matter; CP, crude protein; EE, ether extract; CF, crude fiber; NDF, neutral detergent fiber; ADF, Acid detergent fiber; ST, starch.

**Table 2:** Chemical composition of 5 wheat bran samples (%, DM basis)

Items	Experiment 2					Experiment 3	Mean	CV (%)

	Wheat bran 1	Wheat bran 2	Wheat bran 3	Wheat bran 4	Wheat bran 5			
DM	88.10	87.90	87.20	88.80	88.10	87.70	87.97	0.60
CP	18.04	17.23	18.17	18.30	18.02	17.868	17.94	2.10
EE	4.43	4.31	4.43	4.20	4.31	4.33	4.34	2.00
CF	14.07	14.30	14.19	13.96	14.07	14.03	14.10	0.86
NDF	48.01	48.58	48.69	46.77	48.47	49.03	48.26	1.66
ADF	15.32	16.00	15.44	15.78	16.91	15.85	15.88	3.55
ST (g/kg)	149.83	145.29	150.23	143.02	140.75	161.92	148.51	5.09

CV, coefficient of variation; DM, dry matter; CP, crude protein; EE, ether extract; CF, crude fiber; NDF, neutral detergent fiber; ADF, Acid detergent fiber; ST, starch.

**Table 3:** Ingredient composition of the reference and test diets (air-dry basis, %)

Items	Reference diet	Test diet
Corn	60.82	41.96
Soybean meal	21.42	14.78
Corn gluten meal	2.60	1.79
Peanut meal	3.00	2.07
Soybean oil	4.50	3.10
DDGS	3.00	2.07
Test wheat/wheat bran	0.00	30.00
L-lysine HCl, 70%	1.00	0.69
D, L-methionine, 99%	0.25	0.17
L-threonine, 99%	0.14	0.10
Monocalcium phosphate	1.14	1.14
Salt	0.25	0.25
Sodium humate	0.20	0.20
Choline chloride, 60%	0.11	0.11
Sodium bicarbonate	0.12	0.12
Calcium Propionate	0.02	0.02
Coarse stone	0.90	0.90
Vitamin and mineral premix^1)^	0.50	0.50
L-trptophan	0.03	0.03
Total	100.00	100.00

DDGS, dried distillers grains with solubles.

1) Premix provided the following per kg of the diet: vitamin A 12,500 IU; vitamin D_3_ 3,500 IU; vitamins E 20 IU; vitamin K 3 mg; vitamin B_1_ 0.01 mg; vitamin B_2_ 8.00 mg; vitamin B_6_ 4.5 mg; vitamin B_12_ 0.02 mg; nicotinic acid 34 mg; pantothenic acid 12 mg; folic acid 0.5 mg; biotin 0.2 mg; Fe 80 mg; Cu 8 mg; Zn 80 mg; Mn 80 mg; I 0.7 mg; Se 0.3 mg.

**Table 4:** Nutrient levels of the reference diet and test diets (air-dry basis, %)

Items	Experiment 1	Experiment 2											Experiment 3	
	
		Reference diet	Wheat 1	Wheat 2	Wheat 3	Wheat 4	Wheat 5	Wheat bran 1	Wheat bran 2	Wheat bran 3	Wheat bran 4	Wheat bran 5	Wheat	Wheat bran
DM	88.26	93.63	92.84	94.25	93.81	92.60	93.99	93.44	92.74	92.40	94.27	95.25	90.10	92.10
GE	16.94	18.27	17.68	17.76	17.79	17.85	17.52	17.80	17.62	17.75	17.86	18.05	18.39	17.28
CP	20.65	21.04	18.34	19.96	19.53	20.43	20.10	20.42	20.46	20.11	20.60	20.98	20.06	20.59

DM, dry matter; GE, gross energy; CP, crude protein.

**Table 5:** Effect of wheat diet composition on performance and energy in broilers

Items	Experiment 1		Experiment 2								Experiment 3	
		
	Mean	RSD	Reference diet	Test diet					SEM	p-value	Mean	RSD

				Wheat 1	Wheat 2	Wheat 3	Wheat 4	Wheat 5				
Growth performance												
ABW (g)	462.16	5.18	414.34	440.66	450.63	429.38	425.96	407.04	4.46	0.20	445.21	2.53
FI (g DM/d)	64.16	7.37	56.37^ab^	51.43^a^	71.08^c^	68.27^bc^	65.59^bc^	56.29^ab^	2.16	0.02	59.38	6.25
ADG (g/d)	62.52	15.85	38.64	42.50	43.58	37.86	45.08	31.86	1.66	0.24	32.08	12.16
FCR (g/g DM)	1.04	11.67	1.53	1.48	1.65	1.82	1.46	1.83	0.07	0.51	1.86	7.97
Energy balance (MJ/kg BW^0.70^/d)												
AME intake	1.62	5.45	1.38^a^	1.34^a^	1.66^b^	1.58^b^	1.57^b^	1.27^a^	0.03	<0.01	1.35	6.04
NE intake	1.13	8.83	0.85^ab^	0.77^a^	1.02^c^	1.02^c^	1.00^bc^	0.78^a^	0.03	0.05	0.91	6.44
THP	0.94	11.23	0.98	1.02	1.09	1.02	1.03	0.94	0.01	0.75	0.89	3.50
HI	0.49	21.49	0.53^a^	0.57^ab^	0.64^b^	0.57^ab^	0.58^ab^	0.49^a^	0.01	0.18	0.44	6.22
RE	0.68	14.7	0.41^ab^	0.32^a^	0.57^c^	0.57^c^	0.55^bc^	0.33^a^	0.03	0.05	0.46	15.39
RQ	0.99	3.49	1.00	1.01	0.98	0.98	0.99	0.99	<0.01	0.46	0.94	4.04
Energy values (MJ/kg DM)												
AME	14.72	1.65	13.29	12.55	12.4	12.91	12.63	12.49	0.25	0.93	12.94	1.63
AMEn	14.28	1.61	13.06	12.35	12.16	12.63	12.37	12.24	0.24	0.92	12.34	3.07
NE	10.26	8.17	8.20	7.22	8.05	8.33	8.47	7.60	0.18	0.32	8.65	2.68
Energy utilization (%)												
AME/GE	76.67	1.65	72.73	65.91	65.83	59.36	65.54	66.97	1.69	0.65	70.39	1.63
AMEn/GE	74.38	1.61	71.45	65.8	65.69	59.25	65.39	66.83	1.66	0.84	67.10	3.05
NE/AME	69.71	8.35	62.04	56.84	60.81	60.22	62.95	60.76	0.96	0.58	66.79	1.78
NE/AMEn	71.86	8.38	63.15	57.73	62.21	61.61	64.44	61.99	1.00	0.54	70.04	1.77

RSD, relative standard deviation; SEM, standard error of the mean; ABW, average body weight; FI, feed intake; ADG, average daily gain; FCR, feed conversion ratio; BW, body weight; AME, apparent metabolizable energy; NE, net energy; THP, total heat production; HI, heat increment; RE, retention of energy; RQ, respiratory quotient; AMEn, apparent metabolizable energy corrected to zero nitrogen retention; GE, gross energy.

a–c Means within a row lacking a common superscript differ (p<0.05).

**Table 6:** Effect of wheat bran diet composition on performance and energy in broilers

Items	Experiment 1		Experiment 2								Experiment 3	
		
	Mean	RSD	Reference diet	Test diet					SEM	p-value	Mean	RSD

				Wheat bran 1	Wheat bran 2	Wheat bran 3	Wheat bran 4	Wheat bran 5				
Growth performance												
ABW (g)	462.16	5.18	414.34^a^	362.32^ab^	368.65^ab^	354.59^b^	358.69^b^	321.40^b^	6.65	0.01	347.33	2.45
FI (g DM/d)	64.16	7.37	56.37	57.04	56.43	56.42	55.17	50.42	1.11	0.64	53.28	2.53
ADG (g/d)	62.52	15.85	38.64	37.29	35.24	37.56	45.00	34.87	1.69	0.62	38.51	7.02
FCR (g/g DM)	1.04	11.67	1.53	1.72	1.65	1.57	1.28	1.57	0.05	0.28	1.39	9.28
Energy balance (MJ/kg BW^0.70^/d)												
AME intake	1.62	5.45	1.38^a^	1.23^ab^	1.21^ab^	1.19^b^	1.24^ab^	1.20^b^	0.02	0.05	1.23	4.08
NE intake	1.13	8.83	0.86^a^	0.70^b^	0.65^b^	0.60^b^	0.67^b^	0.64^b^	0.02	0.03	0.66	13.68
THP	0.94	11.23	0.98	0.97	1.00	1.04	1.02	1.01	0.02	0.81	1.02	11.31
HI	0.49	21.49	0.53	0.53	0.55	0.59	0.57	0.56	0.02	0.81	0.57	20.23
RE	0.68	14.70	0.41^a^	0.25^b^	0.21^b^	0.16^b^	0.22^b^	0.19^b^	0.02	0.03	0.21	42.53
RQ	0.99	3.49	1.00	0.99	0.98	1.01	0.99	0.99	<0.01	0.54	1.01	2.50
Energy values (MJ/kg DM)												
AME	14.72	1.65	13.29^a^	11.22^b^	10.88^b^	11.05^b^	11.33^b^	11.12^b^	0.20	<0.01	11.04	1.74
AMEn	14.28	1.61	13.06^a^	11.03^b^	10.67^b^	10.88^b^	11.13^b^	10.95^b^	0.20	<0.01	10.89	1.46
NE	10.26	8.17	8.20^a^	6.38^b^	5.72^b^	5.82^b^	5.88^b^	6.07^b^	0.16	<0.01	8.19	13.30
Energy utilization (%)												
AME/GE	0.77	1.65	72.73^a^	58.89^b^	55.81^b^	57.55^b^	59.79^b^	58.73^b^	1.2	<0.01	63.85	1.74
AMEn/GE	0.74	1.61	71.45^a^	58.78^b^	55.70^b^	57.45^b^	59.68^b^	58.63^b^	1.09	<0.01	63.00	1.46
NE/AME	0.70	8.35	62.04	57.07	54.16	52.16	51.29	53.41	0.61	0.13	74.20	14.95
NE/AMEn	0.72	8.38	63.15	58.11	55.26	52.98	52.19	54.25	0.62	0.12	75.22	14.68

RSD, relative standard deviation; SEM, standard error of the mean; ABW, average body weight; FI, feed intake; ADG, average daily gain; FCR, feed conversion ratio; DM, dry matter; AME, apparent metabolizable energy; NE, net energy; THP, total heat production; HI, heat increment; RE, retention of energy; RQ, respiratory quotient; AMEn, apparent metabolizable energy corrected to zero nitrogen retention; GE, gross energy.

a,b Means within a row lacking a common superscript differ (p<0.05).

**Table 7:** AME, AMEn, NE, and energy utilization values of wheat in broilers

Items	Experiment 2							Experiment 3	
	
	1	2	3	4	5	SEM	p-value	Mean	RSD
Energy value (MJ/kg DM)									
AME	12.93	13.10	13.09	13.75	13.32	0.33	0.98	13.79	2.53
AMEn	12.7	12.83	12.81	13.50	13.06	0.32	0.98	13.21	2.47
NE	9.34	10.02	10.27	11.33	10.49	0.23	0.93	10.48	3.90
Energy utilization (% DM)									
AME/GE	70.90	71.33	72.02	73.25	72.47	1.79	0.89	69.71	2.53
AMEn/GE	69.62	69.85	70.52	71.93	71.04	1.75	0.89	66.77	2.47
NE/AME	72.24	76.48	78.51	82.38	78.74	1.91	0.58	75.96	1.68
NE/AMEn	73.57	78.10	80.18	83.89	80.33	1.94	0.58	79.32	1.83

AME, apparent metabolizable energy; AMEn, apparent metabolizable energy corrected to zero nitrogen retention; NE, net energy; SEM, standard error of the mean; RSD, relative standard deviation; DM, dry matter; GE, gross energy.

**Table 8:** AME, AMEn, NE, and energy utilization values of wheat bran in broilers

Items	Experiment 2							Experiment 3	
	
	1	2	3	4	5	SEM	p-value	Mean	RSD
Energy value (MJ/kg DM)									
AME	7.72	7.09	6.97	6.93	6.85	0.21	0.72	6.90	9.00
AMEn	7.49	6.88	6.79	6.73	6.66	0.21	0.74	6.75	9.20
NE	5.37	5.17	4.87	5.06	4.88	0.18	0.95	4.82	7.89
Energy utilization (% DM)									
AME/GE	60.62	58.46	59.6	57.21	58.11	1.65	0.98	61.16	5.50
AMEn/GE	58.88	56.79	58.02	55.49	56.58	1.64	0.98	59.79	5.50
NE/AME	69.61	72.98	69.83	72.93	71.27	2.58	0.99	70.01	5.54
NE/AMEn	71.67	75.12	71.73	75.19	73.2	2.66	0.99	71.63	5.77

AME, apparent metabolizable energy; AMEn, apparent metabolizable energy corrected to zero nitrogen retention; NE, net energy; SEM, standard error of the mean; RSD, relative standard deviation; DM, dry matter; GE, gross energy.

**Table 9:** Correlation between nutrient parameters of wheat used for the NE value prediction

Item	CP	EE	CF	NDF	ADF	ST	GE	AME	AMEn	NE
CP	1.00									
EE	0.16	1.00								
CF	−0.16	0.43^*^	1.00							
NDF	−0.14	0.27	0.98^**^	1.00						
ADF	0.29	−0.27	0.53^**^	0.64^**^	1.00					
ST	0.37^*^	0.84^**^	0.03	−0.13	−0.29	1.00				
GE	−0.23	0.17	−0.12	−0.19	−0.05	0.50^**^	1.00			
AME	−0.14	0.42^*^	−0.18	−0.31	−0.31	0.73^**^	0.94^**^	1.00		
AMEn	−0.11	0.43^*^	−0.14	−0.27	−0.25	0.74^**^	0.94^**^	0.99^**^	1.00	
NE	−0.14	0.45^*^	−0.35	−0.49^**^	−0.57^**^	0.74^**^	0.81^**^	0.96^**^	0.94^**^	1.00

NE, net energy; CP, crude protein; EE, ether extract; CF, crude fiber; NDF, neutral detergent fiber; ADF, Acid detergent fiber; ST, starch; GE, gross energy; AME, apparent metabolizable energy; AMEn, apparent metabolizable energy corrected to zero nitrogen retention.

*,** represent p<0.05 and p<0.01, respectively.

**Table 10:** Correlations between nutrient parameters of wheat bran used for the NE value prediction

Item	CP	EE	CF	NDF	ADF	ST	GE	AME	AMEn	NE
CP	1.00									
EE	0.18	1.00								
CF	−0.36	0.71^**^	1.00							
NDF	−0.16	0.82^**^	0.92^**^	1.00						
ADF	−0.24	−0.26	0.02	0.22	1.00					
ST	0.19	0.79^**^	0.50^**^	0.41^*^	−0.79^**^	1.00				
GE	−0.21	0.03	−0.30	−0.36^*^	−0.57^**^	0.34	1.00			
AME	−0.08	0.44^*^	−0.01	−0.01	−0.61^**^	0.63^**^	0.91^**^	1.00		
AMEn	−0.06	0.46^*^	0.00	0.01	−0.62^**^	0.64^**^	0.90^**^	1.00^**^	1.00	
NE	−0.41^*^	0.05	−0.13	−0.26	−0.55^**^	0.37^*^	0.97^**^	0.89^**^	0.87^**^	1.00

NE, net energy; CP, crude protein; EE, ether extract; CF, crude fiber; NDF, neutral detergent fiber; ADF, Acid detergent fiber; ST, starch; GE, gross energy; AME, apparent metabolizable energy; AMEn, apparent metabolizable energy corrected to zero nitrogen retention.

*,** represent p<0.05 and p<0.01, respectively.

**Table 11:** Prediction of NE (MJ/kg DM basis) of wheat and wheat bran from ingredient composition and ME content (MJ/kg DM basis)

Items	Equation No.	Energy (MJ/kg DM)	Equation 1									R^2^

			Intercept	AME	AMEn	CP	EE	CF	ADF	NDF	ST	
Wheat	1	NE	−14.227	1.968	-	-	-	-	−0.411	-	-	0.999
Wheat bran	2		20.87	-	-	−0.362	-	−0.382	−0.244	-	-	0.785

NE, net energy; DM, dry matter; ME, metabolizable energy; AME, apparent metabolizable energy; AMEn, apparent metabolizable energy corrected to zero nitrogen retention; CP, crude protein; EE, ether extract; CF, crude fiber; ADF, acid detergent fiber; NDF, neutral detergent fiber; ST, starch.
